# The Combination of IgA and IgG autoantibodies against Transcriptional Intermediary Factor-1γ contributes to the early diagnosis of Lung Cancer

**DOI:** 10.7150/ijms.47463

**Published:** 2020-06-21

**Authors:** Lili Yu, Xiaoqing Lin, Liangming Zhang, Qingwei Wu, Songgao Zhang, Dunyan Chen, Xiaojie Pan, Yi Huang

**Affiliations:** 1 Provincial Clinical College, Fujian Medical University, Fuzhou 350001, China.; 2 Department of Clinical Laboratory, Fujian Provincial Hospital, Fuzhou 350001, China.; 3 Department of Thoracic Surgery, Fujian Provincial Hospital, Fuzhou 350001, China.; 4 Center for Experimental Research in Clinical Medicine, Fujian Provincial Hospital, Fuzhou 350001, China.

**Keywords:** transcriptional intermediary factor-1γ, autoantibody, lung cancer, early diagnosis, combined detection

## Abstract

**Objective:** The aberrant expression of tumor-associated antigens (TAAs) is responsible for the release of large amounts of autoantibodies in sera, and serum autoantibody detection has been demonstrated to contribute to the early diagnosis of malignancies. Recent studies showed the closely correlation of transcriptional intermediary factor-1γ (TIF1γ) with some malignancies. In our pilot study, we found aberrantly high expression of TIF1γ protein existed in cancer tissues other than matched paracancerous tissues of patients with lung cancer (LC) at early stage by immunohistochemistry (IHC) staining. As a result, this study aims to detect the expression of autoantibodies against TIF1γ in sera of patients with LC at early stage by using enzyme-linked immunosorbent assay (ELISA) and investigate its potential value for the early diagnosis of LC.

**Methods:** The expressions of TIF1γ protein in 60 pairs of LC tissues and matched paracancerous tissues were detected by IHC staining. The levels of anti-TIF1γ-IgA, IgG, IgM, and IgE in the sera of 248 patients with LC at early stage, 200 patients with lung benign lesions (LBL), and 218 healthy controls (HC) were detected by ELISA, respectively. Western blot was used to validate the ELISA results of serum autoantibodies against TIF1γ.

**Results:** The positive rate of TIF1γ protein expression in LC tissues was 83.33%, which was significantly higher than 25.00% in paracancerous tissues (*P*<0.01). The levels and positive rates of serum anti-TIF1γ-IgM and anti-TIF1γ-IgE in early LC group had no significant difference from that in LBL group and HC group (*P*>0.05), while the levels and positive rates of anti-TIF1γ-IgA and anti-TIF1γ-IgG were significantly higher than that in LBL group and HC group (*P*<0.01), of which anti-TIF1γ-IgA showed the area under the receiver operating characteristic curve (AUC) of 0.704 for the patients with LC at early stage, with 28.20% sensitivity at 95.93% specificity, and anti-TIF1γ-IgG showed the AUC of 0.622 for the patients with LC at early stage, with 18.54% sensitivity at 94.25% specificity. The results of anti-TIF1γ-IgA and anti-TIF1γ-IgG in western blot were consistent with that in ELISA. Additionally, the combination of anti-TIF1γ-IgA and anti-TIF1γ-IgG improved the AUC to 0.734, with 38.31% sensitivity at 92.34% specificity.

**Conclusions:** There is a strong humoral immune response to autologous TIF1γ existing in patients with early LC. Both serum anti-TIF1γ-IgA and anti-TIF1γ-IgG show the diagnostic value for the patients with LC at early stage, of which anti-TIF1γ-IgA is donstrated to be a preferable biomarker, and the combined detection of anti-TIF1γ-IgA and anti-TIF1γ**-**IgG might contribute to the further improvement of early diagnosis for LC.

## Introduction

Lung cancer (LC) is a malignant tumor with the highest morbidity and mortality worldwide 1-2. Early diagnosis and timely surgical radical resection are of great significance for the prognosis of LC patients. Previous studies have shown that 5-year survival of the patients with LC at TNM I stage who undergo surgery could reach as high as 92% 3-4; however, due to the absence of apparent symptoms at the early stage, most of patients with LC present in the late stage of the disease, missing the best period for surgery, which leads to a dismal 5-year survival rate of 5% - 15% in LC 5. Therefore, improving the efficiency of early diagnosis of LC is important for the patients' prognosis.

Serological biomarkers are convenient and safe in the screening of asymptomatic populations; nevertheless, the current tumor antigen markers, such as CA125, CA199, neuron specific enolase (NSE), carcinoembryonic antigen (CEA), and cytokeratin 19 fragment (CYFRA 21-1) meet a different degree of detection sensitivity or specificity problems, which limit their clinical value for diagnosing LC 6-7. Recent advances revealed that during the tumorigenesis, even a small amount of aberrantly expressed tumor-associated antigens (TAAs) could be responsible for the release of a large number of autoantibodies in sera, which made serum autoantibody detection contribute to the early diagnosis of various malignancies 8-9. Interestingly, it was shown that autoantibodies against TAAs can be detected in the sera of cancer patients 5 years prior to the manifestation of clinical symptoms 10.

Transcriptional intermediary factor-1γ (TIF1γ), also known as TRIM33, ECTO, PTC7, and RFG7, is a regulatory factor of the TGF-β/Smad pathway characterized by E3 ubiquitin-protein ligase activity. Existed studies have shown that TIF1γ could participate in the regulation of cell proliferation, differentiation, migration and invasion by inhibiting Smad4 to regulate the TGF-β signaling pathway 11-12. Over recent years, the relationship between TIF1γ and malignancies has gained increasing attention. For example, Jain et al 13 found that TIF1γ was aberrantly expressed in colon cancer, provided by the evidence that TIF1γ protein expression in cancer tissues was significantly upregulated compared with that in normal intestinal mucosa tissues, and correlated with the progression of malignancy. The aberrantly high expression of TIF1γ was also reported in breast cancer. By the immunohistochemistry (IHC) staining in a cohort of 248 cases with breast cancer, it was shown the positive rates of nuclear TIF1γ and cytoplasmic TGFβ1 in cancer tissues reached up to 35.9% and 30.4%, respectively; additionally, the high expressions of both TIF1γ and TGFβ1 were closely related to the poor prognosis of the patients, including distant metastasis and recurrence 14 . These above studies indicated that the aberrantly high expression of TIF1γ might contribute to the occurrence and development of colon cancer and breast cancer. Nevertheless, as compared with that in colon cancer and breast cancer, TIF1γ expressions were reported to be significantly downregulated in monocytic leukemia and pancreatic cancer 15-16, suggesting TIF1γ might play different roles in the development of different types of malignancies.

Considering the correlation of TIF1γ with some malignancies, whether the aberrant expression of TIF1γ existed in lung cancer is worth exploring. In our pilot study, we demonstrated aberrantly high expression of TIF1γ protein existed in cancer tissues other than matched paracancerous tissues of patients with lung cancer (LC) at early stage by immunohistochemistry (IHC) staining, which indicated that serum autoantibodies against TIF1γ might be promising approach for the early diagnosis of patients with LC. Therefore, in this study, we intended to comprehensively evaluate the diagnostic value of IgA, G, M and E isotypes of autoantibodies against TIF1γ in the patients with LC at early stage by enzyme-linked immunosorbent assay (ELISA).

## Materials and Methods

### Study subjects

248 patients with LC at early stage, 200 patients with benign lung lesions (LBL), and 218 healthy controls (HC) were recruited from September 2016 to October 2018 at Fujian Provincial Hospital. All patients involved in this study were strictly complied with the diagnostic standards recognized by international or professional societies and none of them had accepted any treatment for the malignancy; the clinical and pathological data were listed in Table 1. 218 HC participants in this group received health examinations from the physical examination centre of Fujian Provincial Hospital and showed no evidence of disease, including malignancies, LBL, etc. Each subject was collected 5 ml peripheral blood before the surgery and the serum was separated at 3000 rpm for 5 min and stored at -80°C until use. 60 pairs of LC tissues and matched paracancerous tissues for IHC staining were randomly selected from 248 cases with LC at early stage. This study was approved by the Institutional Review Board of Fujian Provincial Hospital, and all participants provided written informed consent.

### Immunohistochemistry (IHC) staining

Formalin-fixed, paraffin-embedded 3-mm thick sections were deparaffinized and rehydrated. These preparations were stained at room temperature. Staining was performed using an SP immunohistochemistry kit (Fuzhou Maixin Biotech, China), following the manufacturer's recommendations. The sections were, respectively, incubated with primary antibodies against TIF1γ (Cell Signaling Technology ,USA) (1:1000) for 18 h at 4 ℃, followed by incubation with the biotinylated secondary antibody for 10 min at room temperature, and horseradish peroxidase-conjugated streptavidin for 10 min at room temperature. The immnunoreactivities were visualized brown with diaminobenzidine (DAB kit; Lab Vision) and counterstained with Mayer's hematoxylin. The primary antibody was replaced with non-immune sera for negative controls, keeping all other steps in the process the same. Specimens were conducted under identical conditions. The outcome of IHC staining for 60 pairs of LC tissues and matched paracancerous tissues, randomly selected from 148 cases of adenocarcinoma, 64 cases of squamous cell carcinomas and 36 cases of small cell lung cancer, was manually evaluated and scored by two independent certified pathologists. The intensity of staining was graded as: 0=undetectable, 1+=weak staining, 2+=moderate straining, and 3+=strong staining.

### Enzyme-linked immunosorbent assay (ELISA)

GST-tagged recombinant protein, TIF1γ (expressed by yeast and provided by CDI Laboratories, Inc., USA) was coated onto 96-well plates at 50 ng/well at 4 °C overnight, and the nonspecific binding was blocked by 3% bovine serum albumin (BSA). The wells were incubated with serum samples (1:500 dilution for anti-TIF1γ-IgG and anti-TIF1γ-IgM detection, 1:100 dilution for anti-TIF1γ-IgA and anti-TIF1γ-IgE detection) to carry out the standard ELISA assay as described previously 17, and immunoreactivity was measured by reading the A450. All assays were performed in duplicate, and the averaged OD value was calculated.

### Western blot

GST-tagged recombinant proteins, TIF1γ (150 kD) at 200 ng (expressed by yeast and provided by CDI Laboratories, Inc., USA) and the GST protein (26 kDa) at 50 ng were denatured at 99 °C, and subjected to vertical electrophoresis on 8% SDS-PAGE. Subsequently, the separated proteins were electrotransferred onto the nitrocellulose membrane, and the membranes were incubated with serum samples (1:500 dilution for anti-TIF1γ-IgG detection, 1:100 dilution for anti-TIF1γ-IgA detection) or rabbit anti-GST antibody (Cwbiotech, China) to carry out the standard western blot assay as described previously 18. Finally, the immunoreactive bands were visualized by chemiluminescence imager (Shanghai PeiQing, China).

### Statistical analysis

SPSS 22.0 statistical software was used for the analysis of the experimental data. For all autoantibodies, cut-offs based on mean+2.5SD of HC group were used. χ^2^ test was used for the comparison of rates between groups, and Mann-Whitney U-test,a nonparametric test, was used for group comparisons. Graphpad Prism 5 was used to draw charts. Medcalc v18.11 was used to draw ROC curves and AUC values were calculated.* P*<0.05 was considered a significant difference.

## Results

### Expression of TIF1γ protein in early LC tissues

**Figure 1:**
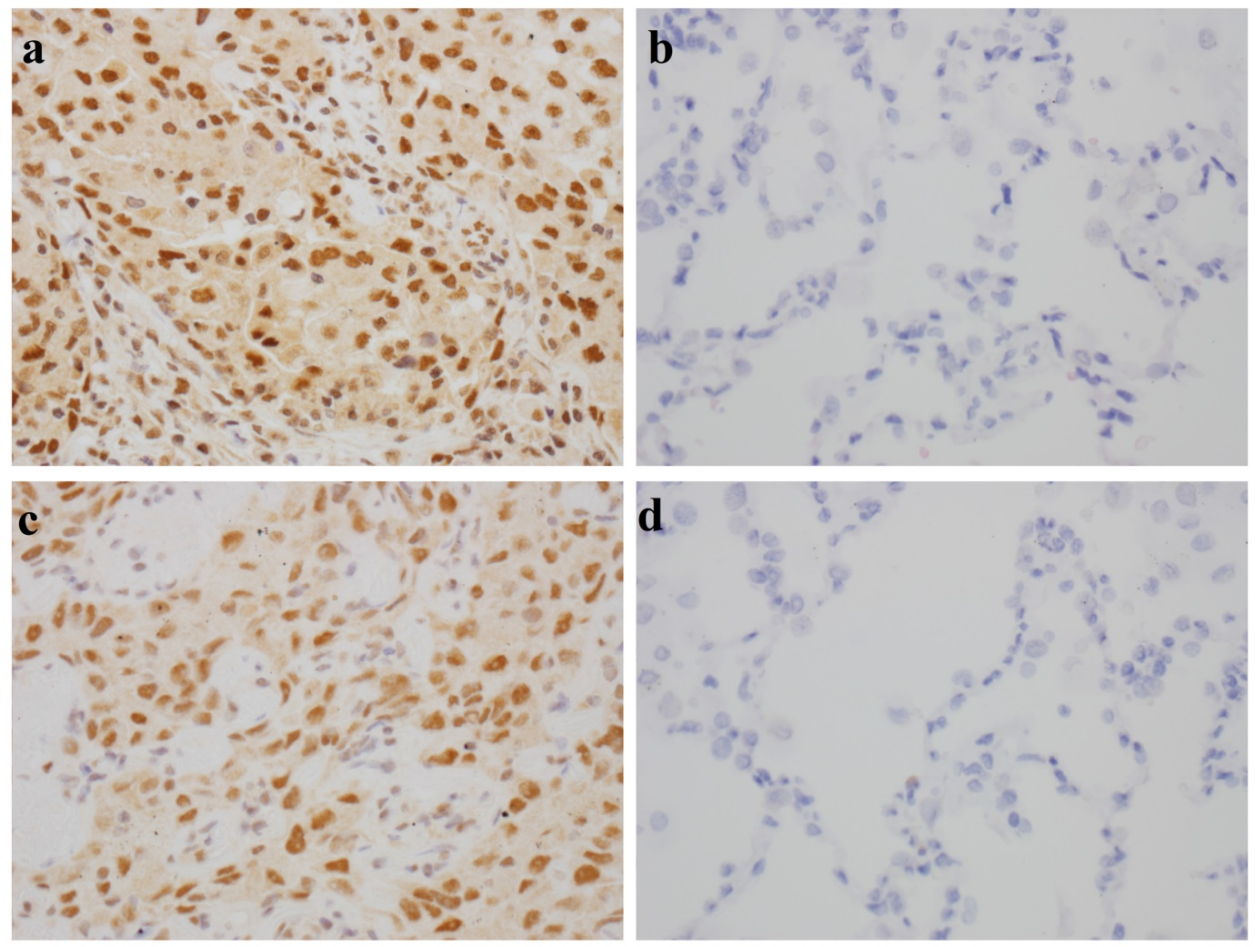
Immunohistochemical staining of TIF1γ in LC tissues and paracancerous tissues (×400). (**A,C**) Strong expression of TIF1γ with 3+, 2+ staining in LC tissues; (**B,D**) Negative or weak expression of TIF1γ in paracancerous tissues.

The results of IHC staining showed that TIF1γ protein was localized in the nucleus and cytoplasm and focally or diffusely distributed brownish yellow or tan-colored granules in LC tissue (**Figure 1A, C**), while it was negative or weakly expressed in matched paracancerous tissue (**Figure 1B, D**). The positive rate of TIF1γ expression in LC tissue was 83.33% (50/60), which was significantly higher than that in matched paracancerous tissue (25.00%, 15/60; *P*<0.01). Besides, there was no significant difference in the positive rates of TIF1γ expression among adenocarcinoma, squamous cell carcinoma and small cell lung cancer (*P*>0.05, **Table 2**).

### Expression of autoantibodies against TIF1γ in sera of patients with early LC

**Figure 2:**
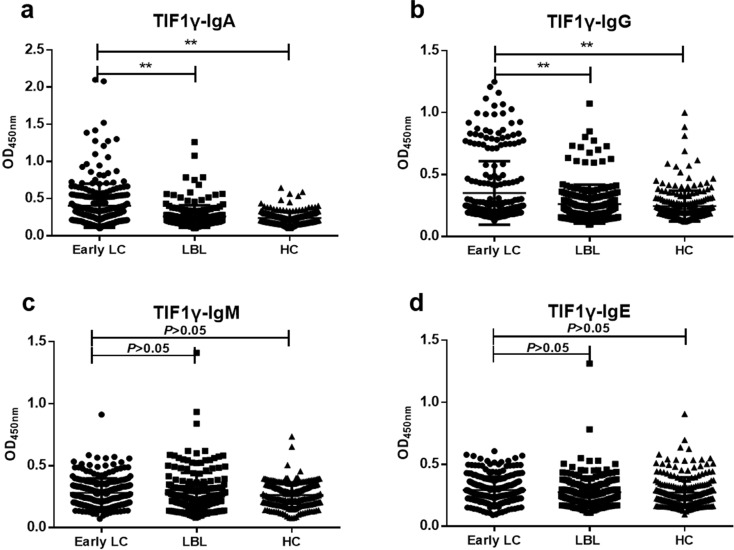
Expression levels of anti-TIF1γ-IgA (**A**), IgG (**B**), IgM (**C**) and IgE (**D**) among three groups. ^**^P<0.01versus Early group.

**Figure 3:**
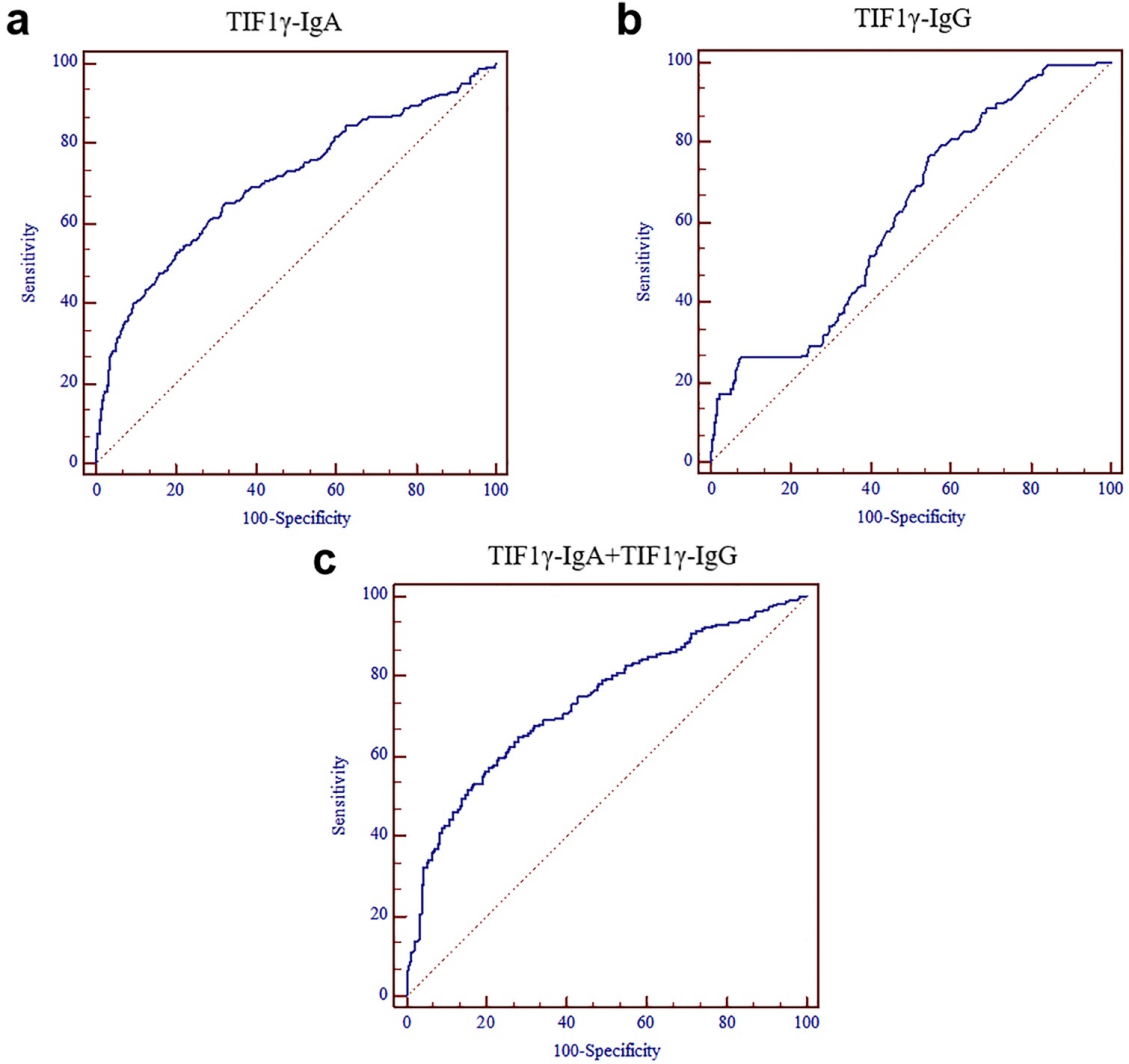
ROC curves of serum TIF1γ-IgA and TIF1γ-IgG for the diagnosis of the patients with LC at early stage. (**A**) ROC curve of serum TIF1γ-IgA; (**B**) ROC curve of serum TIF1γ-IgG; (**C**) ROC curve of the combined detection of serum TIF1γ-IgA and TIF1γ-IgG.

The results of ELISA showed that the levels of anti-TIF1γ-IgA and anti-TIF1γ-IgG in early LC group were significantly higher than that in LBL group and HC group (*P*<0.01, **Figure 2A, B**), while there was no significant difference in the expression of anti-TIF1γ-IgM and anti-TIF1γ-IgE among three groups (*P*>0.05, **Figure 2C, D**). The AUC of anti-TIF1γ-IgA for the patients with early LC was 0.704, with 28.20% sensitivity at 95.93% specificity, and the AUC of anti-TIF1γ-IgG for the patients with early LC was 0.622, with 18.54% sensitivity at 94.25% specificity. Additionally, the AUC of the combination of anti-TIF1γ-IgA with anti-TIF1γ-IgG for the patients with early LC was 0.734, with 38.31% sensitivity at 92.34% specificity (**Table 3**, **Table 4**, **Figure 3**).

### Western blot validation of ELISA results

**Figure 4:**
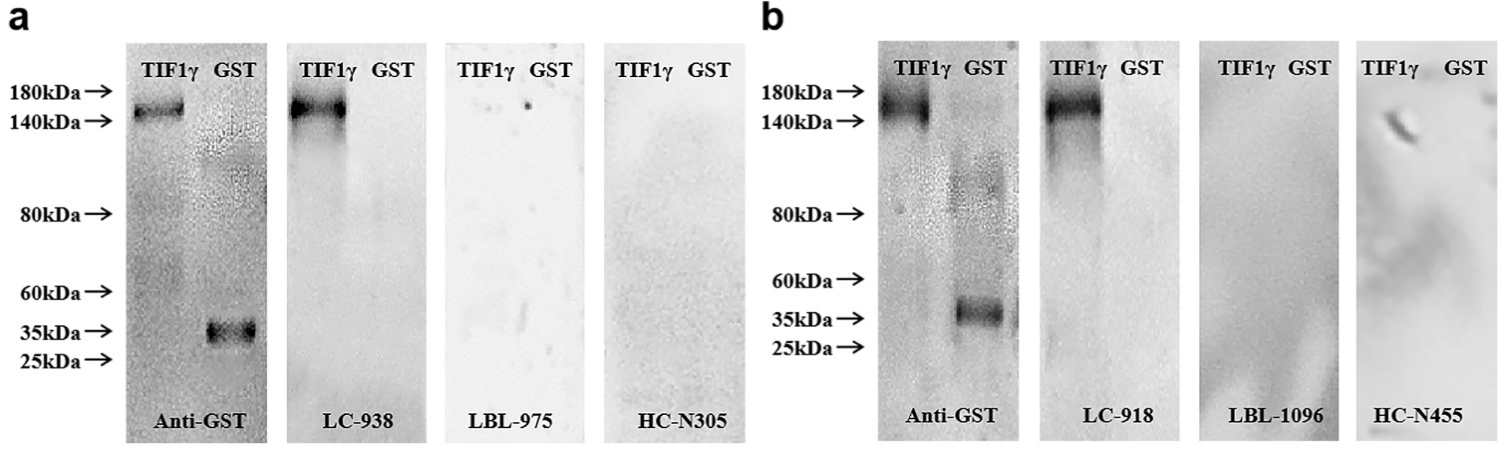
Western blot results of serum TIF1γ-IgA (**A**) and TIF1γ-IgG (**B**).

GST-tagged recombinant protein TIF1γ expressed in yeast was detected by western blot to validate the serum reactivity observed in ELISA. As shown in **Figure 4A**, **B**, the serum of early LC patients with anti-TIF1γ-IgA (+) and anti-TIF1γ-IgG (+) detected by ELISA only bound to the target protein, and not to GST protein; the serum of LBL patients and HC with anti-TIF1γ-IgA(-) and anti-TIF1γ-IgG (-) detected by ELISA did not bind to the target protein or GST protein.

## Discussion

An increasing number of studies have represented that the aberrant expression of TAAs could induce the humoral immune response for TAAs and release autoantibodies into sera during the tumorigenesis. Detecting the serum autoantibodies against TAAs contributes to distinguishing the patients with malignancies from healthy control 19. Even though the level of TAAs is very low, the immune system can still detect the presence of TAAs, producing a large quantity of autoantibodies, which participate to a certain extent in amplification of the antigen signals. Therefore, the autoantibody detection has demonstrated superior diagnostic sensitivity for various malignancies at early stage to the antigen markers 19-20; additionally, the TAA-associated autoantibody presents a long half-life and high persistence in sera for quite a long period, and is easy for the detection 21. These excellent characteristics of autoantibodies against TAAs make it possible to serve as the promising serological markers for the diagnosis of patients with early LC.

By the detection of expression of TIF1γ protein in 60 pairs of LC tissues and matched paracancerous tissues using IHC staining, the positive rate of TIF1γ protein in LC cancer tissues was shown 83.33% (50/60), which was significantly higher than 25.00% (15/60) in matched paracancerous tissues (*P*<0.01). Hence, TIF1γ is demonstrated to be highly expressed in LC tissues at the protein level; nevertheless, the high expression of TIF1γ protein in our study was inconsistent with the poor expression of TIF1γ mRNA expression of TIF1γ in Wang et al study 22, which might be related to the posttranscriptional modification of TIF1γ mRNA existed in LC tissues. On basis of high expression of TIF1γ protein in the cancer tissues, it is reasonably elucidated that TIF1γ is responsible for the release of autoantibodies against TIF1γ in sera of patients with LC at early stage. Therefore, it is worth exploring whether serum autoantibodies against TIF1γ is promising approach for the early diagnosis of patients with LC. And then, we performed the comprehensive evaluation of the diagnostic performances of anti-TIF1γ-IgA, IgG, IgM, and IgE in a cohort of up to 666 serum samples of 248 patients with LC at early stage, 200 patients with LBL, and 218 HC. Our results demonstrated the high potentials of IgA and IgG other than IgM and IgE autoantibodies against TIF1γ for the diagnosis of early LC, of which anti-TIF1γ-IgA presented the preferable diagnostic value for the patients with early LC. It was shown the levels and positive rates of serum anti-TIF1γ-IgM and anti-TIF1γ-IgE in early LC group had no significant difference from that in LBL group and HC group (*P*>0.05), while the levels and positive rates of anti-TIF1γ-IgA and anti-TIF1γ-IgG were significantly higher than that in LBL group and HC group (*P*<0.01), of which anti-TIF1γ-IgA presented the AUC of 0.704 for the patients with LC at early stage, with 28.20% sensitivity at 95.93% specificity, and anti-TIF1γ-IgG presented the AUC of 0.622 for the patients with LC at early stage, with 18.54% sensitivity at 94.25% specificity. Additionally, we further validated the existence of a large quantity of IgA and IgG autoantibodies against TIF1γ in the sera of patients with early LC by the evidence that results of western blot were consistent with that of ELISA assay.

In view of the sensitivity of individual detection of serum anti-TIF1γ-IgA and anti-TIF1γ-IgG for early diagnosis of LC is relatively limited, we performed the further evaluation of combination of IgA and IgG autoantibodies against TIF1γ. Interestingly, it was shown that the diagnostic performance for early LC was further improved by this combined detection compared with individual autoantibody detection, supported by improving AUC to 0.734, with up to 38.31% sensitivity at 92.34% specificity for the patients with early LC.

In conclusion, to the best of our knowledge, this study was the first to discover the strong humoral immune response to autologous TIF1 existed in patients with early LC. Both serum IgA and IgG autoantibodies against TIF1γ present the diagnostic value for the patients with LC at early stage, of which anti-TIF1γ-IgA is demonstrated to be a preferable biomarker, and the combined detection of anti-TIF1γ-IgA and anti-TIF1γ**-**IgG might contribute to the further improvement of early diagnosis for LC. Finally, it is essential further studies should be carried out to unearth more valuable IgA and IgG autoantibodies to enchance the sensitivity at high specificity for the diagnosis of early LC, so as to further improve the early diagnostic efficiency and 5-year survival rate of LC.

## Figures and Tables

**Table 1:** Clinical Characteristics of the Early LC, LBL and HC groups

Variable	Early LC (n = 248)			LBL (n = 200)			HC (n = 218)			*P*
	No.	Mean	%	No.	Mean	%	No.	Mean	%	
**Age (years)**										0.07
Mean		59.7			57.9			57.9		
Standard deviation		10.4			11.0			8.8		
**Sex**										0.550
Male	170		68.6	132		66.0	139		63.8	
Female	78		31.4	68		34.0	79		36.2	
**Smoking history**	139		56.0	99		49.5	105		48.2	0.188
**Type**										
AD	148		59.7							
SCC	64		25.8							
SCLC	36		14.5							
**Stage**										
I	164		66.1							
II	48		19.4							
Limited-stage	36		14.5							
**LBL**										
Pneumonia				106		53.0				
Pulmonary nodule				52		26.0				
COPD				22		11.0				
Pulmonary tuberculosis				20		10.0				

**Abbreviations:** AD: Adenocarcinoma; SCC: Squamous Cell Carcinoma; SCLC: Small Cell Lung Cancer; COPD: Chronic obstructive pulmonary disease.

**Table 2:** Comparison of positive rates of TIF1γ expression between early LC tissues and paracancerous tissues

	n	-	1+	2+	3+	Positive rate (%)	*P*
Early LC tissues	60	10	13	14	23	83.33 (50/60)	0.000
Paracancerous tissues	60	45	15	0	0	25.00 (15/60)	
**Type**							0.634
AD	32	4	6	9	13	87.5 (28/32)	
SCC	20	4	5	4	7	80.0 (16/20)	
SCLC	8	2	2	1	3	75.0 (6/8)	

**Table 3:** Comparison of positive rates of anti-TIF1γ expression among Early LC, LBL and HC groups

	Early LC	LBL	HC	Specificity (%)
	(n=248)	(n=200)	(n=218)	
	Sensitivity (%)	Sensitivity (%)	Sensitivity (%)	
TIF1γ-IgA	28.20(70/248)^**^	6.50(13/200)	1.83(4/218)	95.93(401/418)
TIF1γ-IgG	18.54(46/248)^**^	7.50(15/200)	4.12(9/218)	94.25(394/418)
TIF1γ-IgM	4.84(12/248)	9.50(19/200)	1.38(3/218)	94.74(396/418)
TIF1γ-IgE	4.45(11/248)	3.50(7/200)	6.89(15/218)	94.74(396/418)
TIF1γ-IgA+TIF1γ-IgG	38.31(95/248)^**^	10.50(21/200)	5.04(11/218)	92.34(386/418)

**P<0.01 versus LBL/HC groups.

**Table 4:** Comparison of the performance of Serum TIF1γ-IgA and TIF1γ-IgG in diagnosing the patients with LC at early stage

	AUC	SE	95% CI	*P*
TIF1γ-IgA	0.704	0.0217	0.668 - 0.739	<0.0001
TIF1γ-IgG	0.622	0.0219	0.584 - 0.659	<0.0001
TIF1γ-IgA +TIF1γ-IgG	0.734	0.0205	0.699 - 0.768	<0.0001

## Abbreviations

LC: lung cancer
TIF1γ: transcriptional intermediary factor-1γ
LBL: lung benign lesions
HC: healthy controls
TAAs: tumor-associated antigens
AD: Adenocarcinoma
SCC: Squamous Cell Carcinoma
SCLC: Small Cell Lung Cancer
COPD: Chronic obstructive pulmonary disease
